# Outcomes from different minimally invasive approaches for infected necrotizing pancreatitis

**DOI:** 10.1097/MD.0000000000016111

**Published:** 2019-06-14

**Authors:** Yong Hu, Xun Jiang, Chunyan Li, Yunfeng Cui

**Affiliations:** a Tianjin Medical University; b Department of Surgery, Tianjin Nankai Hospital, Nankai Clinical School of Medicine, Tianjin Medical University, Tianjin, China.

**Keywords:** infected necrotizing pancreatitis, minimally invasive surgery, primary endpoints, secondary endpoints

## Abstract

Infected necrotizing pancreatitis (INP), the leading cause of mortality in the late phase of acute pancreatitis, nearly always requires intervention. In recent years minimal invasive surgery is becoming more and more popular for the management of INP, but few studies compared different minimally invasive strategies. The objective of this observation study was to evaluate the safety and effectiveness with several minimal invasive treatment.

We retrospectively reviewed cases of percutaneous catheter drainage (PCD), minimal access retroperitoneal pancreatic necrosectomy (MARPN), small incision pancreatic necrosectom (SIPN), single-incision access port retroperitoneoscopic debridement (SIAPRD) for INP between January 2013 and October 2018. Data were analyzed for the primary endpoints as well as secondary endpoints.

Eighty-one patients with INP were treated by minimally invasive procedures including PCD (n = 32), MARPN (n = 18), SIPN (n = 16), and SIAPRD (n = 15). Overall mortality was greatest after PCD 34% (MARPN 11% vs SIPN 6% vs SIRLD6%). Problems after initial surgery were ongoing sepsis (PCD 56% vs MARPN 50% vs SIPN 31% vs SIAPRD13%; *P* < .05). There was a significant difference in number of interventions (median, 6 vs 5 vs 3 vs 2; *P* < .05). Time from onset of symptoms to recovery was less for SIAPRD than for PCD, MARPN, or SIPN (median, 45 vs 102 vs 80 vs 67 days; *P* < .05).

SIAPRD remedy evidently improved outcomes, including systemic inflammatory response syndrome, number of interventions, length of hospital stay and overall cost. It is technically feasible, safe, and effective for INP, in contrast to others, and can achieve the best clinical results with the least cost. Furthermore, relevant multicentre randomized controlled trials are eager to prove these findings.

## 1 Introduction

Acute necrotic collections and walled-off necrosis, often performed at least 2 weeks after the onset of symptoms,^[1]^ become infected in about one-third of patients.^[2]^ It is associated with higher mortality rate of up to 30% and an acknowledged indication for surgical intervention.^[3]^ It occurs only in moderately severe or severe acute pancreatitis (AP) and remains a major public health burden with over 300,000 hospitalizations/yr in the United States, accounting for the second highest cost of hospital stays (2.5 billion dollars).^[4]^ Early aggressive fluid resuscitation, enteral nutrition, antibiotics, and intervention are of vital importance to treat Infected necrotizing pancreatitis (INP).^[1,5]^ Surgical methods and timing are the focus of controversy in the treatment of INP.^[6]^ Traditionally, laparotomy was the only tool available for surgical treatment of pancreatic necrosis.^[7]^ But several clinical evidence was found to be associated with high rate of prolonged multi-organ failure, mortality, and result in local complications such as bleeding, gastrointestinal fistula, reoperations, as well as a high rate of postoperative diabetes, mainly due to the deterioration of general condition and the serious damage to the abdominal structure and pancreatic tissue.^[8]^ Therefore minimal invasive techniques have been developed to reduce surgical stress, and thereby limit its deleterious influence on patient's condition. The use of minimally invasive techniques, such as percutaneous catheter drainage (PCD) and minimal access retroperitoneal pancreatic necrosectomy (MARPN) has gained increasing popularity in a few medical centers.^[9]^ At present, most scholars believe that the intervention should be delayed to about 4 weeks later, and more clinical evidence is needed to confirm it. Several cohort studies on necrotizing pancreatitis have been published over the past decades, 88 patients reported in Hjalmar C's randomized controlled trial (RCT) underwent minimally invasive approach which reduced the rate of major complications or mortality compared with open surgery.^[10]^ Ninety-eight patients included in van Brunschot's RCT show neither major complications nor mortality differed between the 2 groups, although fistulas were less common and hospital stays were shorter in the endoscopy arm.^[11]^ Although these studies were methodologically sound, they included highly selective patients, most of whom had no organ failures. In clinical practice, organ failures are common in patients with IPN. In short, minimally invasive approach being more preferable than open surgery has become an expert consensus, but the best of minimally invasive interventions are currently not cleared. Over the past 5 years, our experienced pancreatic multidisciplinary group has embraced several novel minimally invasive approaches to NP treatment with great breakthroughs. In order to evaluate the best operation, we conducted a retrospective study of the patients treated in our surgery center, by comparing the treatment results with the various surgical intervention, hoping to find out the most ideal minimally invasive approach in INP.

## 2 Patients and methods

Eighty-one consecutive patients with a diagnosis of INP admitted to Nankai University Nankai Hospital who underwent either PCD, MARPN, small incision pancreatic necrosectom (SIPN), or single-incision access port retroperitoneoscopic debridement (SIAPRD) between January 2013 and October 2018 were identified retrospectively and included in this cohort analysis. Twenty-nine cases had been treated at outside facilities for 4 to 30 days but their condition gradually deteriorated and was complicated by hypovolemia, hypoxemia, and high fever, so they were transferred to our hospital. All patients were managed by a dedicated pancreatic team consisting of interventional endoscopists, critical care surgeons, dedicated interventional radiologists, hospitalists, and dieticians. Decisions for intervention were made by this team, and reviewed on an ongoing basis at a weekly conference. Exclusion criteria: postsurgical AP, AP as a secondary diagnosis. The study was approved by the Ethics Committee of the Nankai University Nankai Hospital (NKYY_YX_IRB_2018_002_01). Written informed consent was obtained from all patients or their parents before surgery. AP was defined according to the 2013 revision of the Atlanta classification as an association of 2 of the 3 following features: typical abdominal pain (acute onset of a persistent, severe, epigastric pain often radiating to the back), serum lipase or amylase activity at least 3 times greater than the upper limit of normal, and characteristic findings of AP on abdominal cross-sectional imaging studies.^[12]^ INP has a mortality of 30%, which can be diagnosed in 3 ways:

(1) by gas configurations in the necrotic collection on imaging,
(2) by a positive gram stain or culture from a (percutaneous) fine-needle aspiration of the necrotic collection or
(3) suspected by clinical diagnosis.

Clinical suspicion of infection is based on signs of infection (temperature >38.5°C, rising serum inflammatory markers) or when new/persistent organ failure occurs, which is typically most reliable after the initial phase of SIRS.^[13]^ Sepsis was defined according to the 1992 ACCS/SCCM criteria.^[14]^ Contrast-enhanced abdominal computed tomography (CT) showed the area of infected necrosis, including the lesser sac, left or right anterior pararenal space, retroduodenal space, and left or right paracolic gutters. It is convenient for assessing the severity of the disease and next surgery.

### 2.1 Surgical protocol

**Figure 1:**
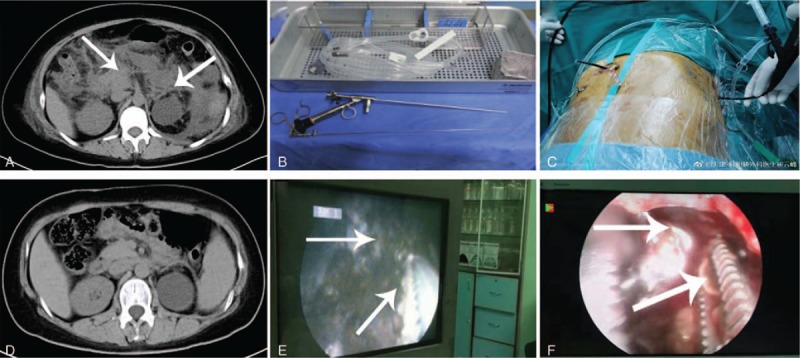
(A) Computed tomography before the first necrosectomy; (B and E) percutaneous nephroscopic device and video-guided necrotic tissue removal; (C and F) cholangioscopy-guided necrotic tissue removal; (D) computed tomography after last necrosectomy.

MARPN: Under general anesthesia, the catheter is exchanged over a guide wire for serial renal dilators and the track dilated to 30 Fr. An operating choledochoscope or nephroscope with a wide-bore operating channel (initially Wolf, later Storz) is then used to access the necrosis, and if necessary, a combination of soft-mirror and hard-lens was used. Normal saline is quickly flushed into the pus cavity through the water injection hole, and pus is vacuumed out by negative pressure suction function repeatedly. If solid necrotic tissue attachment is found on the wall of pus cavity under video, it can be removed by piecemeal with video-assisted biopsy forceps. Samples of the removed necrosis are sent for microbiological examination. Following initial debridement, a multifunctional irrigating drain (M10), consisting of a porous outer sleeve and an inner core that can be attracted by vacuum, is inserted into the cavity and 0.9% saline solution used to irrigate the cavity continuously at a rate of 125 mL/h (Figure 1).

**Figure 2:**
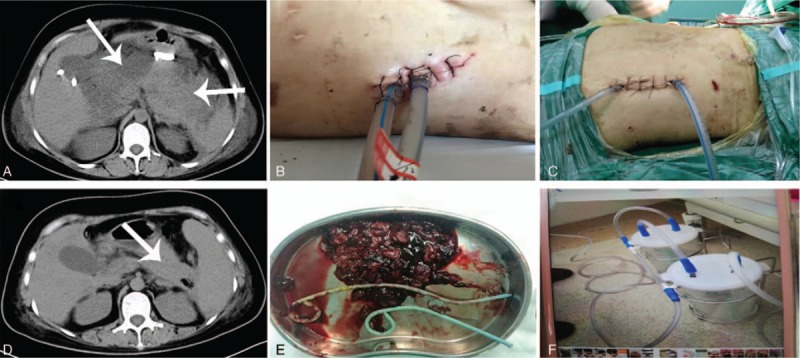
(A) Computed tomography before the first necrosectomy; (B and C) small incision minimally invasive approach; (D) computed tomography after last necrosectomy; (E) infected necrotic tissue; (F) negative pressure flushing device.

SIPN: The position of the access was determined under CT guidance. Generally, when the infection necrosis area is located at the bilateral retroperitoneal space of the colon, incision is carried out near the mid-axillary line of the bilateral lumbocostal region, while the necrotic infection area is located in the peripancreatic space and the lesser omental sac, the nearest point to the skin is taken for incision. Make a small incision of about 2 to 5 cm at the abdominal wall of the drainage tube, and dissect the skin, subcutaneous, muscle, and fascia layer by layer. During the whole process, the sinus path formed by the PCD drainage tube was used to enter the abscess cavity (which could greatly reduce the possibility of gastrointestinal side-injury caused by the mistake into the abdominal cavity). A large number of necrotic tissues within the cavity were found under direct vision and then removed with noninvasive forceps or manual. The peripancreatic collection is reached through the space between the spleen, the left kidney, and the descending colon. The peripancreatic area is accessed by pushing aside the posterior parietal peritoneum and the colon towards the midline, taking the left kidney as a reference. The necrotic tissue was removed and then the pus cavity was washed with normal saline to confirm that there was no residual necrotic tissue. As far as possible, multiple multifunctional flushing drainage tubes (M10) were inserted into the cavity, and removing necrotic tissue by positive pressure irrigation with 0.9% saline solution and continuous negative pressure suction. If there are multiple infectious foci of pancreatic necrosis, multiple mini-incisions can be used to debride necrotic tissue (Figure 2).

**Figure 3:**
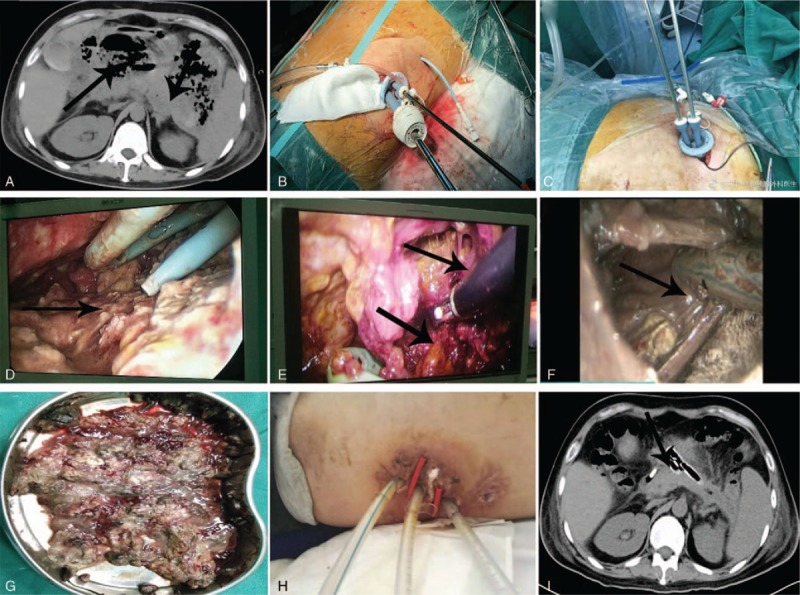
(A) Computed tomography before the first necrosectomy; (B, C, D, E, and F) single-incision access port retroperitoneoscopic debridement; (G) infected necrotic tissue; (H) minimally invasive incision; I: computed tomography after last necrosectomy.

SIAPRD: Under general anesthesia, we use a single access port placed through a short incision in the left lumbar region. The patient was placed in the lateral position (60°) with the affected side facing upward, and fixed with position frames. Necrotic collections are accessed through the left retroperitoneum with a small incision of about 2 to 3 cm at the abdominal wall of the drainage tube mouth, establish pneumoperitoneum by pneumoperitoneal needle puncture. This port is a flexible soft foam device with access channels for 3 cannulas. One 12-mm trocar and 2 5-mm trocars were passed through the port. The first trocar was placed with a laparoscope inside for visual guidance. The other trocars were then inserted under laparoscopic guidance from within the necrotic cavity. Necrosectomy was performed using a 5-mm laparoscope and 5-mm instruments. The perirenal fascia was opened along the front of the psoas muscle from the retrocolic space, and then the retroperitoneal space was opened along the top of the pancreatic tail until the lesser sac. Using warm saline positive pressure rinse and negative pressure aspirator to aspirate necrotic pus. Single-hole forceps were used to grasp the solid necrotic tissue attached to the abscess, which was not easy to wash. Stop the operation and suture the incision when the abscess wall turns pink and there is no residual necrotic tissue or active hemorrhage. Our technique of retroperitoneoscopic pancreatic necrosectomy using the single-access port has several advantages. This technique allows safe placement of all working trocars under visual guidance and secure establishment of gas insufflation. The visualization of the necrotic cavity can be achieved using optional gas insufflation or continuous saline irrigation. Two additional instruments can be used simultaneously with a laparoscope. A 12-mm trocar allows the removal of large pieces of necrotic material and efficacious lavage of the necrotic cavity. The necrosis located in the lesser sac can be easily approached with this technique (Figure 3).

### 2.2 Data collection

The electronic records of all patients treated at our institution were searched for the ICD code of AP from January 2013 to October 2018 so as not to miss any patients with INP. All patients who underwent minimally invasive treatment were identified by OPS codes and patient records reviewed. For analysis, 4 groups were defined: PCD, MARPN, SIPN, and SIAPRD.

The following data were collected from the electronic files and patient charts: patient characteristics (age, gender, body mass index [BMI], coexisting conditions), pancreatitis characteristics (etiology, Balthazar score, American Society of Anesthesiologists [ASA] class, CT severity index, disease severity), The primary endpoint was a composite of major complications consist of treatment success, new-onset multiple organ failure, persistent systemic inflammatory response syndrome (SIRS), pancreatic-cutaneous fistula, intra-abdominal bleeding and perforation of a visceral organ, and death during 3 months of follow up. Secondary endpoints included pancreatic endocrine and exocrine insufficiency, Incisional hernia, number of interventions, length of hospital stay, days in intensive care unit (ICU), overall cost, and the pancreatic endocrine and exocrine insufficiency of 1 year after the treatments.

### 2.3 Statistical analysis

For the statistical analysis, SPSS 22.0 software was used. The Kolmogorov–Smirnov test was used to assess whether continuous data were normally distributed. The results are presented as mean and standard deviations, as numbers and percentages or as median and percentiles when confidence intervals were too high. The qualitative variables were analyzed with the chi-squared test and the continuous variables with the Kruskal–Wallis *H* test or the Fisher exact test. Differences with *P* < .05 were accepted as statistically significant.

## 3 Results

**Figure 4:**
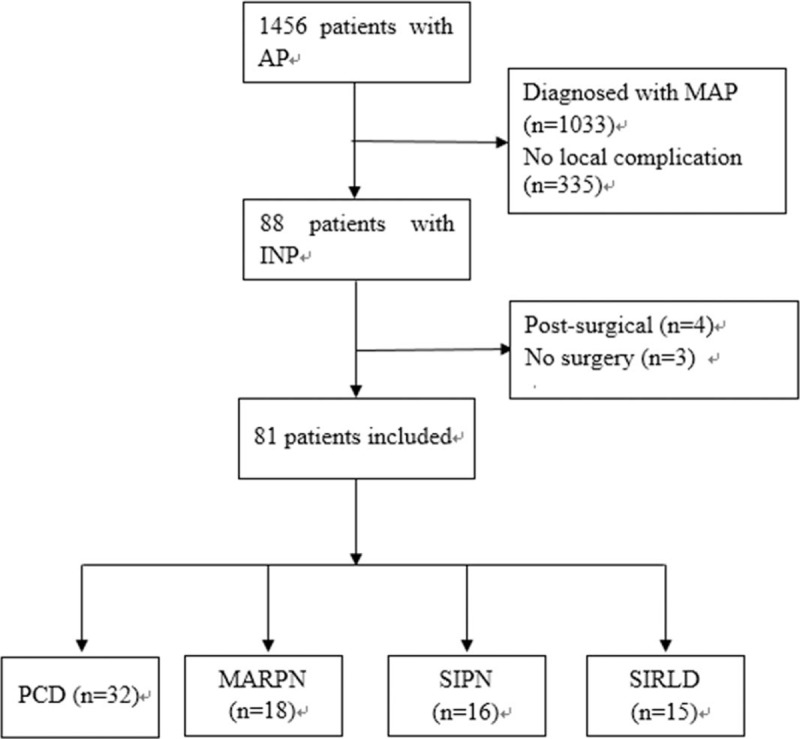
Flow diagram for selection of patients for included in this study.

Figure 4 is the patient flowchart. Of the 1456 patients admitted to our hospital for AP, 1033 were diagnosed with mild acute pancreatitis. Only 88 patients met the diagnostic criteria for INP. Three patients did not undergo surgical treatment. Details of the 81 subjects were included in the study. Mean age was 45 years (range 24–80) with a gender ratio of 1.3 (46 men and 35 women). The commonest etiologies in this series were biliary (n = 32, 39%) and alcohol (n = 18, 22%).

Table 1 shows the baseline characteristics and disease severity for patients undergoing intervention, subdivided in 4 different minimally invasive approaches. No significant differences were found among the 4 groups regarding patient features. As compared with patients treated PCD, patients undergoing MARPN, SIPN, and SIAPRD had higher ASA class, acute physiology and chronic health evaluation II score, C reactive protein, white blood cell count, CT severity index, single organ failure. But there were no significant differences in SIRS and multiple organ failure between the 4 groups.

**Table 1:**
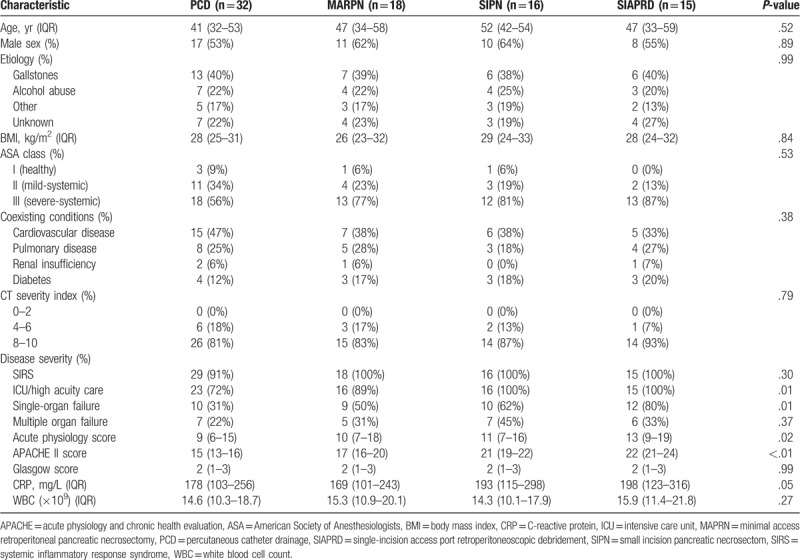
Characteristics of all patients with necrotizing pancreatitis.

Table 2 lists primary endpoints and secondary endpoints for the several treatment approaches. In-hospital mortality was 18% overall, PCD has the greatest mortality (34%) but there was no significant difference between other approaches (MARPN, 11%; SIPN, 6%; SIAPRD, 6%). However, new onset organ failure occurred more frequently in the PCD (25%) and MARPN (22%) group. There was a significant difference in the improvement of sepsis after primary surgery (PCD 56% vs MARPN 50% vs SIPN 31% vs SIAPRD13%; *P* < .05). We observed no significant difference in pancreatic fisture, abdominal bleeding, and visceral perforation among groups. Both ICU and Hospital stays were significantly longer in the patients undergoing PCD and MARPN for INP separately. Patients in the PCD, MARPN, and SIPN groups required more number of interventions and higher treatment cost than SIAPRD (*P* < .05). At 12-month follow-up, we observed no differences regarding exocrine, endocrine insufficiency.

**Table 2:**
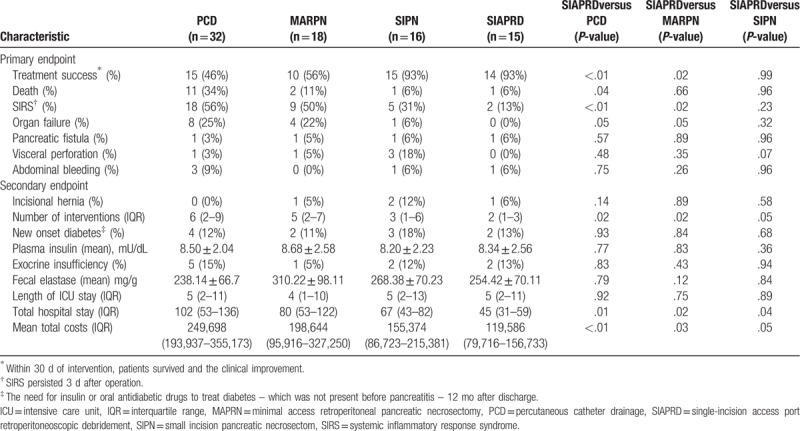
Clinical outcome of all patients with necrotizing pancreatitis.

## 4 Discussion

Infection of necrosis occurred in approximately 30% of patients with necrotizing pancreatitis.^[15]^ It is a heterogeneous disease with a high mortality rate.^[16]^ Therefore treatment must be individualized to specific patient characteristics, including necrosis distribution and size. Since Freeny et al first reported the use of PCD in the treatment of INP,^[17]^ minimally invasive and endoscopic approach has gradually become the primary therapy in the management of INP.^[18]^ And a recent meta-analysis of prospective studies of endoscopic approach in INP, demonstrated no remarkable superiority in the primary outcome compared with minimally invasive approach.^[19]^ As yet, an ideal intervention has not been defined. There are few other studies that have directly compared different minimally invasive necrosectomy. Our multidisciplinary group consists of expert interventional radiologists and pancreatic surgeons. We used multiple approaches to NP treatment in this contemporary period. Mortality in infected necrosis in our study was 18%. This seems to be lower than the mortality of approximately 30% for infected necrosis reported in reviews of the literature of the past 2 decades.^[20]^ Mainly because we have improved and developed some new minimally invasive technologies.

This is a nonrandomized retrospective review of a prospectively maintained database and as such, there were potential select biases. It represents our initial experience with minimally invasive techniques in patients with INP, and thus partially covers the stage of a learning curve. Selection criteria for both SIAPRD and conversion to other therapies in our series were, to a certain degree, subjective and biased. Minimization of these biases was undertaken by using 2 simultaneous approaches: reporting consecutive cases, analysis by intention to treat. First, this study included a large number of patients in 5 years, was consecutive conducted in our single center setting, and covered the entire clinical spectrum of necrotizing pancreatitis. We analyzed all consecutive patients with INP and analyzed clinical data of all patients who met the inclusion criteria. There is no follow-up data lost. 81 patients with a gender ratio of 1.3 (46 men and 35 women) ranged in age from 24 to 80 and came from all over the country. Over the period of the study, we have deliberately chosen to treat as many patients as possible using a minimal access approach. Therefore, the samples included in the study are representative and beneficial to the study results. For a long time, we tried to improve PCD by selecting various penetrating sites and expanding the diameter of the puncture tube for continuous convection washing, but the clinical effect is not significant. Later, we began to try to improve the method of debridement surgery. SIPN, which we have improved on the basis of open surgical treatment, has minimal trauma, few local complications and high efficiency in removing necrotic tissue. This technique mostly removes necrotic tissue from the anterior abdominal cavity or retroperitoneum through a small incision along the PCD tube tract. Over the time period of this study, we found a necrotic resection method (SIAPRD) that is more novel and effective than SIPN. SIAPRD (a newest minimally invasive technique) was associated with fewer complications (organ failure, SIRS), and the use of SIAPRD instead of PCD, MARPN, and SIPN as the first-line surgery method used to treat IPN was associated with shorter ICU and hospital stays and with shorter times on nutrition and hemodynamic support which may reflect an efficiency and safety greatly increased in removal of necrotic infection. And this method can maximally improve the systemic inflammatory state and minimize the trauma to the patient. SIAPRD is very convenient to operate and has a clear field of vision. It can remove necrotic tissue from all peripancreatic sites to the greatest extent under the condition of safety. Maybe that is why the treatment outcomes are better than the other 3 groups.

These results support that the significant decreased systemic and local inflammation with SIAPRD compared with other minimally invasive procedures, effectively control of infected necrotic lesions, thereby avoiding the need for multiple surgeries. In the other 3 groups, about 60% of patients need more than 2 necrotic resections to recover. Although endoscopic necrosis has been accepted, applied sparingly in our institutional experience, likely reflecting highly select indications, as well as liberal use of surgical transgastric debridement. In the past year, increasing number severe SAP has come to our center for treatment. We prefer SIAPRD to treat patients and observe the superiority of this method. So far, only 1 patient has died, and length of hospital and hospitalization costs of all patients have been greatly reduced. We are conducting some prospective studies to further confirm the clinical efficacy of SIRLD. In our practice, SIAPRD acts a crucial therapeutic role in NP patients. Importantly, these approaches are individualized based on specific patient characteristics including necrosis distribution, physiologic condition, and failure to progress after other treatment. Over this time period we witnessed a significant increase in the number of unsuccessful NP patients treated with PCD and MARPN; the relatively high number that still require effective debridement (>50%) likely reflects the highly complex nature of patients referred to our tertiary center. These complex clinical scenarios include patients who have failed other therapies, as well as those with pancreatic head necrosis and necrosis tracking down paracolic gutters and the root of the small bowel mesentery. This report compares the characteristics and clinical outcomes of different treatments in our surgical center and assess the best treatment options. From the current results analysis, SIAPRD is a very effective and safe method, and it is necessary to widely promote it. SIAPRD can significantly improve the cure rate of INP and reduce hospitalization costs and time. At the same time, it should be noted that necrotizing pancreatitis is a complex and heterogeneous disease. We are supposed to treat patients individually according to the degree of disease progression and the anatomical distribution of necrotic foci. Minimally invasive surgery is only a means, not a constant. Many patients will require more than 1 modality to effect disease resolution, and operative debridement continues to play an important role in management of these patients. Evaluation by a multidisciplinary treatment team composed of experienced gastroenterologists, surgeons, and interventional radiologists is crucial for treatment planning and to achieve optimal patient outcomes. One patient in the SIAPRD group died of abdominal bleeding on the 5th day after surgery. He was 82 years old, obese (BMI = 36), and had hypercholesterolemia, hypertension, and chronic cardiac failure. After 2 weeks of treatment in the external hospital, he continued to have high fever and shortness of breath, and transferred to the surgical center of our hospital for further treatment. At the time of admission, the patient had multiple venous thrombosis in both lower extremities, abnormal coagulation function, and the risk of surgery was extremely high. After communicating with the family, he decided to undergo surgical treatment. On the 5th day after the SIRLD, the patient developed abdominal bleeding and died after emergency laparotomy.

Our study also has limitations. First, this is a retrospective study with a small sample size that utilized direct medical record, and while follow-up was assessed for the present time, it might be prone to selection bias, favoring the results of the intervention. However, sicker patients are generally referred for SIRLD rather than being subjected to surgery as the first-line treatment approach. Second, this is a single center study, which typically offers different therapies, our results may not represent other healthcare settings with differing patient demographics and procedural preferences, such as percutaneous retroperitoneal nephrostomy. Third, transgastric drainage is seldom used in our center. Last but not least, a caveat is in order; however, as SIAPRD and SILD may have been used preferentially in the sickest patients during the late period that may be bias in analyzing the prognosis of patients.

## 5 Conclusion

In summary, SIAPRD has obvious advantages in the treatment of INP, it is safe and effective and can greatly reduce hospitalization time and cost. This study was retrospective and the sample size was small. So there is a great need for more RCTs to confirm these advantages. In addition, future studies will be required to further define the optimal time for the SIAPRD procedure.

## Author contributions

**Data curation:** Chunyan Li.

**Formal analysis:** Yong Hu.

**Methodology:** Xun Jiang.

**Supervision:** Yunfeng Cui.

**Validation:** Chunyan Li.

**Writing – original draft:** Yong Hu.

**Writing – review and editing:** Yong Hu.
